# Religiosity and Sexual Initiation Among Hispanic Adolescents: The Role of Sexual Attitudes

**DOI:** 10.3389/fpsyg.2021.715032

**Published:** 2021-11-05

**Authors:** Maria Calatrava, Carlos Beltramo, Alfonso Osorio, Martiño Rodríguez-González, Jokin De Irala, Cristina Lopez-del Burgo

**Affiliations:** ^1^Institute for Culture and Society, University of Navarra, Pamplona, Spain; ^2^Navarra Institute for Health Research (IdiSNA), Pamplona, Spain; ^3^School of Education and Psychology, University of Navarra, Pamplona, Spain; ^4^Department of Preventive Medicine and Public Health, School of Medicine, University of Navarra, Pamplona, Spain

**Keywords:** religiosity, sexual initiation, sexual attitudes, adolescents, sexual permissiveness

## Abstract

**Introduction:** Religiosity and sexuality present numerous interconnections. Little is known regarding the specific causal pathways between each religiosity dimension and sexual behavior. The objectives of this study were (1) to explore the relationship between religiosity (measured through attendance at religious services, salience, and prayer) and sexual initiation in adolescents and (2) to establish the role of sexual permissiveness as mediator of the impact produced by religiosity in sexual initiation.

**Methods:** This study analyzes data from an ongoing school-based international study examining what young people feel and think about relationships, love, and sexuality. An anonymous, self-administered online questionnaire was developed in Spanish. A total of 4,366 students, aged 14–18, completed the questionnaire. A final sample of 2,919 questionnaires was analyzed. Two unconditional logistic regression models were fit with religiosity variables and possible confounders as independent variables (with and without permissiveness, respectively). The dependent variable was sexual initiation. A final path analysis was performed to further understand the results.

**Results:** Our study highlights that, in predominantly Catholic and Spanish-speaking countries, the fact of attending church and praying may greatly contribute to postponing sexual relations during adolescence, even independently of their attitudes on sexual permissiveness. Conversely, the effect of salience on sexual initiation seems to be fostered only through the mediation of sexual permissiveness. Our findings point to an indirect effect of the three religiosity dimensions (and in particular, religious salience) through permissive attitudes.

**Conclusion:** Religiosity could be a relevant factor to explain sexual initiation during adolescence.

## Introduction

Religiosity is a basic dimension of socialization (Pearce, 2015; Hayward and Pearce, 2021). In Regnerus’s (2007) words, “religion builds youths’ social and organizational ties through social capital, network closure, and extra-community skills” (p. 45). It is receiving growing interest from fields such as public health (Long et al., 2019; Schnitker et al., 2019) and spirituality (Brelsford et al., 2011; Hardy et al., 2019). Several studies underline the role of religiosity in adolescent health, both physical and mental (Twenge et al., 2015; Mills et al., 2017; Pazhoohi et al., 2017), as well as in the development of pro-social behaviors (Good and Willoughby, 2006; Shariff et al., 2016).

Sexuality is a core part of people’s lives and of their health and bio-psycho-social wellbeing (Twenge et al., 2015; Boislard et al., 2016; Soller et al., 2017). Several studies find a relationship between religiosity and sexual activity during adolescence (Rostosky et al., 2004; Štulhofer et al., 2011; Luquis et al., 2012, 2015), showing that sexual initiation begins at later ages among religious adolescents (Scott et al., 2006; Sinha et al., 2007; Gold et al., 2010; Smith, 2015).

Religiosity and sexuality have numerous interconnections (Stolz et al., 2013). Some researchers have suggested that religiosity can impact people’s behavior through the personal assumption of the moral principles of a religion and/or through social influence from their religious social group of reference (Hardy et al., 2019). The theory of reasoned action (TRA) (Fishbein and Ajzen, 1975, 2010; Montaño and Kasprzyk, 2008) has been used by several researchers to explain the link between religiosity and sexual behavior (Regnerus, 2007; Hull et al., 2011; Zhang et al., 2015). Additionally, religiosity has various dimensions (affiliation, attendance, salience, and frequency of prayer) that may have different types of impact on sexual behavior (Sinha et al., 2007).

### Adolescence and Sexual Behavior

Adolescence is a period of physical and psychological development, which is also recognized as a time of mastery for each adolescent’s unique talents, strengths, skills, and interests (Damon, 2004). However, it should be noted that adolescents have been considered a vulnerable group, mainly due to their lack of cognitive and emotional maturity for making reasonable decisions related to their health; this may lead adolescents to engage in risky behaviors such as substance use, delinquency, reckless driving, and sexual risk behaviors (Steinberg, 2008; Kipping et al., 2012; World Health Organization [WHO], 2017).

One of the transitions to adult life is sexual initiation. Sexual experience is frequent among adolescents (Romero-Martínez et al., 2013; Instituto Nacional de la Juventud Chile, 2015; Moreno et al., 2016; Kann, 2019); however, some early sexual activities are considered risk behaviors. For example, penile-vaginal intercourse may lead to known risks, though some of them can be reduced through preventive measures (Makenzius and Larsson, 2013; Boisvert et al., 2017).

Though some controversy exists, there is a certain scientific consensus that delaying the onset of sexual activity may be a beneficial recommendation to protect adolescent health (Halperin et al., 2004; Centers for Disease Control and Prevention, 2019). Consequently, some preventive programs pursue this goal, preferably in conjunction with other preventive measures and strategies for the promotion of positive youth developmental outcomes. It seems advisable to identify the factors associated with sexual initiation during adolescence.

### Religiosity and Its Dimensions

Numerous studies suggest that religiosity has a significant influence on adolescent behavior (Salas-Wright et al., 2017; Holmes et al., 2019; James and Ward, 2019). However, according to some authors, “If researchers use only one domain of religiosity, they likely will fail to capture the collective influence of religiosity on risky sexual behavior” (Smith, 2015, p. 51). In this regard, no consensus has been reached as to which dimensions better define religiosity (King and Boyatzis, 2015) and which of them have greater weight on behaviors such as sexual activity.

One of the first classifications of the dimensions of religiosity was made by Allport and Ross (1967), who distinguished between extrinsic and intrinsic religious motivations. This classification has endured over the years, though other authors have referenced them in terms of public and private religiosity dimensions (Nonnemaker et al., 2003). Nowadays, the more well-known public or extrinsic dimensions are affiliation and attendance. Affiliation has been defined as the acknowledgment of being a follower of a religion or claiming to be religious (King and Boyatzis, 2015), whereas, attendance is defined as physically attending the services offered by a religious group (Krause et al., 2017; Malinakova et al., 2018; King et al., 2019; Pawlikowski et al., 2019).

The private or intrinsic dimension of religiosity includes salience (Salas-Wright et al., 2012, 2017; Moulin-Stożek et al., 2018). This dimension considers the importance a person gives to their religious beliefs in their daily life, especially when it comes to decision-making (Wilt et al., 2017; Mendolia et al., 2018). Consistent with Fishbein and Ajzen (1975, 2010), it is not just a matter of having some religious beliefs, but of recognizing that these beliefs have enough relevance to determine attitudes and conditioning intentional behaviors. “It captures the individual beliefs chosen by the youths, rather than behaviors that could potentially be imposed, or at least affected, by parents and society and their respective expectations” (Mendolia et al., 2018, p. 1).

Another relevant dimension of religiosity is the frequency of prayer, which analyzes the time one dedicates to praying (Scott et al., 2006; Pryor et al., 2007; Luquis et al., 2012). This is a behavioral dimension, rather than referring to an internal construct, and is often practiced with other people (within the family, in the temple, etc.). This would imply that prayer is understood as an extrinsic dimension of religiosity with social features (Krause and Hayward, 2013; Buch Møller et al., 2020). However, prayer can be practiced in private, and can be understood as a personal decision, which would make it an intrinsic dimension (Laird et al., 2004). We can therefore classify prayer as a religious dimension with both intrinsic and extrinsic characteristics (Ladd and Spilka, 2002, 2006).

### Religiosity Dimensions and Sexual Behavior

Religiosity has a complex relationship with behavior, and specifically with sexual behavior. As stated above, the TRA has been used to understand these relationships (Regnerus, 2007; Hull et al., 2011). In these cases, attitudes about sex and normative pressure have been stated as two ways of mediation between the different forms of religiosity and sexual behavior. Some studies found that the association between religiosity and sexual behavior was mediated by attitudes about sex (Meier, 2003; Rostosky et al., 2003). But they also found that, after controlling for attitudes, an independent effect remained, this is, religiosity had both an effect via attitudes and an independent effect. This suggests that the causal path may also work through other mechanisms, possibly non-internal variables such as social peer or other kind of influence.

However, as shown above, religiosity is a complex construct consisting of different dimensions. Previous studies conducted in the United States suggest that not all dimensions of religiosity have the same degree of influence on sexual activity (Sinha et al., 2007). What is the role of the different dimensions of religiosity? Does intrinsic and extrinsic religiosity influence through different causal paths? These questions certainly appear to be valid avenues for consideration.

Mott et al. (1996) found that the influence of church attendance on sexual behavior was mediated by peer influence. More recently, Hull et al. (2011) found that the link between attendance and sexual behavior was mediated both by attitudes (regarding whether having sex would be bad/good and unpleasant/pleasant, etc.) and by perceived normative pressure (perceived sexual intercourse among peers and perceived social approval of sexual intercourse). In other words, extrinsic religiosity influenced both attitudes and normative pressure, while both attitudes and normative pressure influenced sexual behavior.

Other studies have found that religious attendance has a weaker impact than salience on sexual attitudes (Jung, 2016) but a stronger impact on sexual behavior (Penhollow et al., 2005; Edwards et al., 2008, 2011). This could reinforce the idea that extrinsic religiosity has an additional and unique pathway, independent of personal attitudes (for example, social influence, as previously mentioned). It seems, therefore, that intrinsic religiosity influences sexual behavior through personal attitudes, while extrinsic religiosity may influence through both attitudes and peer influence.

**FIGURE 1:**
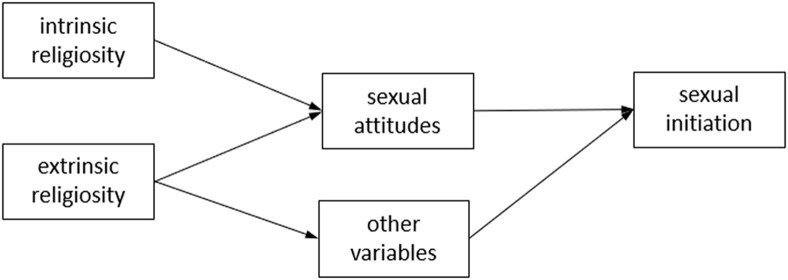
Theoretical model of the relationship between religiosity dimensions and sexual behavior.

Accordingly, following literature on intrinsic/extrinsic religiosity, the TRA, and the mentioned findings in literature, we propose the model shown in Figure 1. The effect of intrinsic religiosity on sexual behavior would be mediated by attitudes, while the effect of extrinsic religiosity would not only be mediated by attitudes, but also by other variables (perceived norms and social pressure…).

### The Mediation Role of Sexual Permissiveness

Within the framework of the TRA, the main mediators in shaping behaviors are attitudes toward behaviors and subjective norms (Fishbein and Ajzen, 1975; Montaño and Kasprzyk, 2008; Ajzen, 2012). As explained above, there is some evidence supporting that religiosity influences sexual behavior through both mediators. In this study we will focus on the role of attitudes.

Sexual attitudes have been considered a key variable to explain sexual behavior, particularly among adolescents (Burris et al., 2009; Hull et al., 2011). These attitudes consist of beliefs, opinions, personal feelings, and perceptions on sexual activity, sexual health, and sexually transmitted infections (STIs) (Cardoza et al., 2012; Alimi, 2019).

Religiosity plays an important role in the forming of sexual attitudes. For instance, Santelli et al. (2004) verified that religiosity can ultimately construct a framework that may act as a relevant factor with regard to sexual initiation. On the other hand, Pluhar et al. (1998) researched the relationship between religiosity and sexual attitudes in university students in the United States. The adolescents that claimed to have guided themselves by their religiosity in their sexual behavior were more likely to show conservative attitudes regarding sexual initiation.

Some of the mentioned studies suggest that both intrinsic and extrinsic religiosity may act on sexual behavior through the mediation of sexual attitudes (Penhollow et al., 2005; Edwards et al., 2008, 2011; Hull et al., 2011; Jung, 2016). However, only partial evidences have been found.

Among sexual attitudes, sexual permissiveness plays an important role (Hendrick and Hendrick, 1987). According to these authors, this construct refers to a casual approach to sexual relationships. Actually, when examining in detail the mentioned studies that assess sexual attitudes, most of them primarily assess sexual permissiveness/restrictiveness: Some focused on possible costs or benefits of having sexual intercourse (Meier, 2003; Rostosky et al., 2004), while others assessed aspects such as approval of premarital sex (Edwards et al., 2008; Jung, 2016).

Studies have found that sexual permissiveness is influenced by different determinants such as age, sex, exposure to pornography, mass media consumption, and religion, among others (Jang and Lee, 2011; Lee and Song, 2016; Dillman Carpentier and Stevens, 2017; Ma, 2018; Jung and Kim, 2020).

### Aims of the Study

Most of the abovementioned studies have been carried out in the United States (Hardy et al., 2019), Europe (Štulhofer et al., 2011; Cranney et al., 2018), or Africa (Alimi, 2019; Francis et al., 2019; Somefun, 2019). In Spanish-speaking countries, where 92% of the population accept that belief in God and is member of any religion (Pew Research Center, 2014), fewer studies are available (Moulin-Stożek et al., 2018; King et al., 2019). Sociologists acknowledge this is a gap in the academic research: “Latin America is a context that has been either ignored or misunderstood in the current debates about the transformation of the religious landscape” (Morello, 2019, p. 9).

In this study, we intend to explore the relationship between the dimensions of religiosity and sexual initiation by including sexual permissiveness as a mediating variable in a sample of Hispanic adolescents. Specifically, we aim: (1) to assess the relationship between intrinsic and extrinsic dimensions of religiosity (attendance at religious services, salience, and prayer), and sexual initiation; and (2) to establish the role of sexual permissiveness as a mediator of the impact produced by religiosity on adolescents’ sexual initiation. Following the literature, and the proposed model (Figure 1), we hypothesize that the three religiosity dimensions will be associated with a delay in sexual initiation (Murray-Swank et al., 2005; Penhollow et al., 2005; Edwards et al., 2008, 2011), and that these associations will be mediated through sexual permissiveness (Hull et al., 2011; Jung, 2016). This mediation will be particularly relevant for intrinsic dimensions (salience and perhaps prayer) (Edwards et al., 2008, 2011; Jung, 2016).

## Materials and Methods

This study analyzes cross-sectional data from the YourLife Project, an ongoing school-based international study examining what young people feel and think about relationships, love, and sexuality.^1^ Details for this project are explained elsewhere (Carlos et al., 2016; Belintxon et al., 2020). Ethical approval for the Project was obtained from the Ethics Committee of the University of Navarra.

### Sample

A total of 52 secondary schools from Spanish-speaking countries participated in the study. For this article, we used data from 4,366 high school students from Spain, Chile, Mexico, and Peru. They were aged between 14 and 18.

A total of 4,366 student participants completed the questionnaire. We eliminated the participants presenting missing values in any of the main study variables (*n* = 1447). A final sample of 2,919 questionnaires was analyzed.

### Instrument and Measures

An anonymous, self-administered online questionnaire was originally developed in Spanish. The questionnaire included questions on adolescents’ lifestyles, environmental influences (family and peers), personal beliefs, opinions, attitudes, and sexual behavior. Below we describe the variables used in this study.

#### Religion and Religiosity-Related Dimensions

Participants were asked “What religion do you practice or believe in?” They then had to select a religious affiliation from a list. This list included the most common religions and the option “other religion.” Adolescents reporting any religious affiliation were also asked about their frequency of church attendance, frequency of prayer, and religious salience.

The frequency of church attendance (extrinsic religiosity) and frequency of prayer (mixed extrinsic/intrinsic religiosity) were measured with the questions “How often do you go to church/temple?” and “How often do you pray?” Response options ranged from 0 (*never*) to 5 (*more than once a week*). Religious salience (intrinsic religiosity) was evaluated using the statement “My faith is an important influence in my life, and I am willing to take it into account in my decisions.” Response options ranged from 0 (*totally disagree*) to 4 (*totally agree*). Each religiosity-related dimension was dichotomized at the median in order to create high and low categorical groups. The adolescents that did not report any religious affiliation were included in the low group for each dimension.

#### Sexual Permissiveness

Sexual permissiveness was measured through three items assessing to what extent the adolescent considers different sexual behaviors appropriate. The items were inspired on the *Illustrative Core Instruments* (Cleland et al., 2001; Potard et al., 2008).

Participants were asked whether they agreed with the following statements: “It is OK for youth my age to have sexual relationships just for fun, without commitment”; “It is better to wait until marriage before having sex”; “Petting (foreplay without full intercourse) among adolescent couples is acceptable”; and, “Having sexual relationships is a need that has to be satisfied (just like eating and sleeping).” Responses were assessed on a five-point Likert scale, from 0 (*totally disagree*) to 4 (*totally agree*). A single measure of *sexual permissiveness* was created, obtaining the mean score of the four items. Internal consistency was acceptable (Cronbach alpha = 0.77; Ordinal alpha = 0.80). This variable was dichotomized at the median to create high and low groups.

#### Sexual Initiation

Sexual initiation was assessed with the question “Have you ever had sexual relations? Remember that by “sexual relations” we are referring to sex with penetration.” Response options included “yes,” “no,” and “I prefer not to answer.” We chose sexual relations with penetration because this sexual behavior is easily distinguishable and identifiable by adolescents, and because it is generally considered the main marker of the transition to heterosexual sexual activity (Carpenter, 2005).

#### Sociodemographic Characteristics

Participants were also asked whether they were girls or boys, and how old they were. We also knew the country where their school was. All these variables were included in the analyses.

### Procedure

Data was collected between 2016 and 2019. Members of the research team and local collaborators in each country invited schools to participate. Information on the project was made available on a web page on which the schools could apply for participation.

Schools willing to participate received a participation link for each grade. Schools were responsible for, and in charge of, obtaining parental consent, in accordance with local laws and policies, under the supervision of the research team. On the participation day and 2 days before, students were given information about the study and were informed that participation was voluntary. Those who were willing to participate accessed the online questionnaire during class hours. Anonymity and confidentiality were protected.

### Data Analysis

Univariate analyses (frequencies and percentages) were used to describe the main characteristics of the sample. Differences between groups of participants were analyzed using χ^2^ tests.

Multivariate unconditional logistic regression models were conducted. Model 1 assessed the association between the dimensions of religiosity and sexual initiation. Model 2 included variables from Model 1 and sexual permissiveness. Both models were adjusted for demographic variables. Odds ratios (OR) and 95% confidence intervals (CI) were estimated. Age was introduced as a continuous variable after testing a linear relationship between age and sexual initiation.

A path analysis was subsequently performed, testing a model in which the religiosity dimensions predict sexual initiation both directly and through the mediation of sexual permissiveness.

Finally, analyses were repeated for Catholics only (since this was, by far, the most frequent religion), and the sample was split by country.

Statistical analyses were performed using STATA version 12.0 for Windows.

## Results

The characteristics of the sample are shown in Table 1. The majority of participants were girls (59.3%). More than 80% were aged 15–17 years old and 85% were Catholics. This proportion was lowest among Chileans (69%) and highest among Mexicans (91%). Around half of the students reported attending religious services (from 37% in Peru to 55% in Spain and Mexico) and praying (from 44% in Chile to 60% in Mexico) at least once a week, but less than 40% considered their faith an important influence in their life (from 34% in Spain to 44% in Mexico). Half of the participants had high sexual permissiveness (from 44% in Mexico to 69% in Chile), and 14.8% had already had sexual intercourse (from 12% in Peru to 20% in Chile). All differences by country were statistically significant (*p* < 0.001).

**TABLE 1:** Characteristics of the sample.

	Chile (*N* = 382)^a^ n (%)	Spain (*N* = 535)^a^ n (%)	Mexico (*N* = 921)^a^ n (%)	Peru (*N* = 1081)^a^ n (%)	Total (*N* = 2919)^a^ n (%)	*p* ^b^
Girl	154 (40.3)	386 (72.1)	500 (54.3)	690 (63.8)	1730 (59.3)	<0.001
**Age**						
14	6 (1.6)	37 (6.9)	97 (10.5)	154 (14.2)	294 (10.1)	<0.001
15	122 (31.9)	239 (44.7)	209 (22.7)	419 (38.8)	989 (33.9)	
16	123 (32.2)	123 (23.0)	298 (32.4)	327 (30.2)	871 (29.8)	
17	67 (17.5)	115 (21.5)	235 (25.5)	131 (12.1)	548 (18.8)	
18	64 (16.8)	21 (3.9)	82 (8.9)	50 (4.6)	217 (7.4)	
**Religion**						
No religious affiliation	77 (20.2)	58 (10.8)	59 (6.4)	87 (8.0)	281 (9.6)	<0.001
Catholic	262 (68.6)	458 (85.6)	839 (91.1)	937 (86.7)	2496 (85.5)	
Other Christian religion^c^	27 (7.1)	8 (1.5)	13 (1.4)	30 (2.8)	78 (2.7)	
Other religions^d^	16 (4.2)	11 (2.1)	10 (1.1)	27 (2.5)	64 (2.2)	
**Weekly church attendance**	178 (46.7)	293 (54.8)	504 (54.8)	397 (37.3)	1372 (47.3)	<0.001
**Weekly prayer**	166 (43.6)	293 (54.9)	551 (60.0)	599 (56.2)	1609 (55.5)	<0.001
**High religious salience^e^**	134 (35.3)	183 (34.3)	410 (44.5)	390 (36.3)	1117 (38.4)	<0.001
**High sexual permissiveness^f^**	258 (69.4)	293 (55.7)	399 (44.4)	497 (47.2)	1447 (50.8)	<0.001
**Sexual initiation**	77 (20.2)	68 (12.7)	161 (17.5)	127 (11.7)	433 (14.8)	<0.001

*^a^Totals vary because some items were not responded by some participants.*

*^b^p value for the Chi-squared test.*

*^c^Other Christian religions include Reformed/Evangelical, Orthodox, and “Other Christian religion.”*

*^d^Other religions include Islam, Buddhism, Hinduism, Jewish, Traditional Chinese religion, and “Other.”*

*^e^Participants answering “I totally agree” to the item: “Do you agree with the following statement? My faith is an important influence in my life, and I am willing to take it into account in my decisions.”*

*^f^Participants over the median in the permissiveness scale.*

When differentiating between girls and boys, prayer was more frequent among girls than boys (58% vs. 51%, *p* < 0.001), while attendance and salience had no significant differences. On the contrary, high sexual permissiveness (44% of girls, 61% of boys, *p* < 0.001) and sexual initiation (11% of girls, 21% of boys, *p* < 0.001) were more frequent among boys.

By age (younger = 14–15 vs. older = 16–18), attendance was more frequent among older than younger participants (50% vs. 44%, *p* = 0.001), while prayer and salience had no significant differences. Furthermore, high sexual permissiveness (54% of olders, 47% of youngers, *p* < 0.001) and sexual initiation (19% of olders, 9% of youngers, *p* < 0.001) were also more frequent among older participants.

As there were very few participants with a non-Christian religion (64 students, 2.2% of the sample), these were eliminated from subsequent analyses.

Students’ sexual permissiveness and sexual initiation, stratified by religiosity variables (affiliation, church attendance, prayer, and salience), are shown in Table 2. All religiosity variables were inversely associated with sexual permissiveness and with sexual initiation (*p* < 0.001 in all cases). Religious salience had the strongest association with sexual permissiveness (φ = 0.35) but the weakest association with sexual initiation (φ = 0.11).

**TABLE 2:** Sexual permissiveness and sexual initiation, by religiosity dimensions.

	High sexual permissiveness			Sexual initiation		
	n (%)^a^	*p* ^b^	Φ^c^	n (%)^d^	*p* ^b^	φ^c^
**Religious affiliation**						
No religion (*N* = 281)	218 (78.7)	<0.001	0.19	78 (27.8)	<0.001	0.13
Christian (*N* = 2574)	1181 (47.1)			331 (12.9)		
**Frequency of church attendance^e^**						
<weekly (*N* = 1483)^f^	921 (63.7)	<0.001	0.28	289 (19.5)	<0.001	0.15
Weekly (*N* = 1372)^f^	478 (35.6)			120 (8.7)		
**Frequency of prayer^g^**						
<weekly (*N* = 1246)^f^	803 (65.8)	<0.001	0.28	258 (20.7)	<0.001	0.16
Weekly (*N* = 1609)^f^	596 (38.0)			151 (9.4)		
**Religious salience^h^**						
Low (*N* = 1738)^f^	1088 (64.1)	<0.001	0.35	302 (17.4)	<0.001	0.11
High (*N* = 1117)^f^	311 (28.6)			107 (9.6)		

*^a^Number (and percentage) of participants with a high mean score (over the median) in sexual permissiveness, measured by the following items: “It is OK for youth my age to have sexual relationships just for fun, without commitment,” “It is better to wait until marriage before having sex,” “Petting (foreplay without full intercourse) among adolescent couples is acceptable,” and “Having sexual relationships is a need that has to be satisfied (such as eating and sleeping).”*

*^b^p value of the χ^2^ test.*

*^c^Effect size (phi coefficient).*

*^d^Number (and percentage) of participants who have had sexual relationships.*

*^e^Answer to the question “How often do you go to the church/temple?”*

*^f^Totals may vary due to missing values in permissiveness or in sexual initiation.*

*^g^Answer to the question “How often do you pray?”*

*^h^Agreement with the sentence “My faith is an important influence in my life, and I am willing to take it into account in my decisions.” “High” salience refers to participants who “totally agree” with the sentence. The remaining participants were classified as having a “Low” salience.*

Unconditional multivariate logistic regressions were conducted to evaluate the adjusted associations between the religiosity dimensions and sexual initiation (Table 3). Model 1 shows a strong association between religiosity dimensions and sexual initiation. Weekly church attendance and weekly prayer were significantly associated with a decrease in the likelihood of sexual initiation (church: OR = 0.43, 95% CI = 0.33–0.56; prayer: OR = 0.60, 95% CI = 0.47–0.78). High religious salience had a similar association with sexual initiation, but was on the limit of statistical significance (OR = 0.77, 95% CI = 0.58–1.00). When sexual permissiveness was added (Model 2), findings were similar to the initial model, but the odds ratios of the religiosity dimensions were attenuated. Both weekly church attendance and weekly prayer maintained significant – though weaker – associations with the outcome (church: OR = 0.57, 95% CI = 0.40–0.69; prayer: OR = 0.68, 95% CI = 0.53–0.89). However, the statistical significance of the association between salience and the outcome was lost (OR = 1.07, 95% CI = 0.81–1.43). Additionally, sexual permissiveness was significantly and strongly associated with the outcome (OR = 4.42, 95% CI = 3.28–5.95).

**TABLE 3:** Association between religious dimensions and sexual initiation.

	Model 1^a^ OR (95% CI)	Model 2^b^ OR (95% CI)
Weekly church attendance (ref: <Weekly)	0.43 (0.33–0.56)	0.53 (0.40–0.69)
Weekly prayer (ref: <Once a week)	0.60 (0.47–0.78)	0.68 (0.53–0.89)
High religious salience (ref: Low)^c^	0.77 (0.58–1.00)	1.07 (0.81–1.43)
**Country (ref: Mexico)**		
Chile	0.91 (0.65–1.26)	0.73 (0.51–1.02)
Spain	0.79 (0.57–1.11)	0.65 (0.46–0.93)
Peru	0.68 (0.52–0.89)	0.69 (0.52–0.91)
Girl (ref = boy)	0.54 (0.43–0.67)	0.64 (0.51–0.82)
Age (years) (continuous; range: 14–17)	1.58 (1.42–1.75)	1.52 (1.37–1.70)
High sexual permissiveness^d^		4.42 (3.28–5.95)

*ref, reference.*

*^a^Odds ratios (and 95% confidence intervals) of sexual initiation, adjusted for all the variables in the table, except for sexual permissiveness.*

*^b^Odds ratios (and 95% confidence intervals) of sexual initiation, adjusted for all the variables in the table.*

*^c^High religious salience means having replied “I totally agree” to the item “My faith is an important influence in my life and I am willing to take it into account in my decisions.”*

*^d^Participants with a high mean score (over the median) in sexual permissiveness, measured by the following items: “It is OK for youth my age to have sexual relationships just for fun, without commitment,” “It is better to wait until marriage before having sex,” “Petting (foreplay without full intercourse) among adolescent couples is acceptable,” and “Having sexual relationships is a need that has to be satisfied (such as eating and sleeping).”*

**FIGURE 2:**
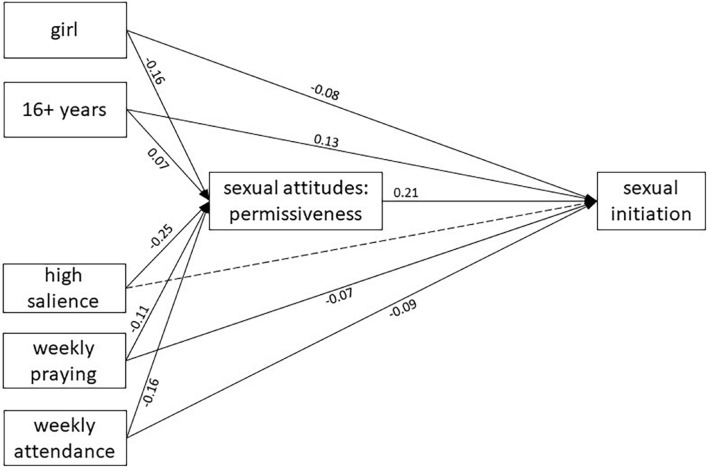
Path analysis of the association between attitudes toward sexual behaviors during adolescence and sexual initiation, considering religiosity dimensions as mediators. Standardized estimates are shown. Solid lines represent significant weights (*p* < 0.001). The dotted line represents a non-significant weight. Errors are omitted for the same reason. The model is saturated and fits perfectly.

Finally, path analysis (Figure 2) supports the hypothesized model, in which the religiosity dimensions predict sexual initiation both directly and through the mediation of sexual permissiveness. The exception is salience, which only presents an indirect, but not direct, effect on sexual initiation.

When all analyses were repeated for Catholics only, or separately by country, results were substantially similar. The differences consisted of lower statistical significance in some cases, probably due to the smaller sample sizes (data not shown).

## Discussion

### Religiosity Dimensions and Sexual Behavior

This study aimed to provide additional evidence on the relationship between religiosity and sexual initiation during adolescence. In particular, we investigated the role of sexual attitudes on that first sexual relationship.

We hypothesized that religiosity could have a substantial role in sexual initiation. As in previous studies conducted in the US (Murray et al., 2007; Regnerus, 2007; Burris et al., 2009; Hull et al., 2011; Luquis et al., 2015), this study found an inverse relationship between religiosity (including church attendance, weekly prayer, and religious salience) and self-reported sexual initiation. Specifically, our three measures of religiosity predicted sexual initiation, with attendance as the best predictor.

This higher predictive power of extrinsic religiosity was already found in previous studies (Penhollow et al., 2005; Edwards et al., 2008, 2011). This has different possible explanations, but we will focus on this one: this dimension of religiosity may act through different pathways, as we will see hereunder.

### The Role of Sexual Permissiveness

Regarding the specific role of sexual permissiveness on the association between religiosity and sexual initiation, we hypothesized a possible mediation: religiosity would decrease sexual permissiveness, which would in turn increase delayed sexual initiation. Do our results support the hypothesis? First, the bivariate results shown in Table 2 support the association between each religiosity dimension and sexual permissiveness, with a higher effect size for religious salience. Second, the association between sexual permissiveness and sexual initiation was subsequently confirmed (Table 3, Model 2): higher levels of sexual permissiveness were related to higher odds of having been sexually active. Third, sexual permissiveness had a certain role on the association between religiosity and sexual initiation: the impact of the religiosity dimensions on the outcome decreased (or even disappeared) by introducing sexual permissiveness into the model (see the differences between both models in Table 3). Specifically, attendance and prayer experienced a slight reduction in their effect, while salience lost its possible effect entirely. Finally, the path analysis (Figure 2) tested the entire model, confirming previous results: the three religiosity variables (and especially salience) reduced sexual permissiveness, which in turn delayed sexual initiation; in addition, religious attendance and praying (but not salience) had a direct effect on sexual initiation, independently of sexual permissiveness.

Therefore, our study comprehensively confirms the partial results of previous studies (Meier, 2003; Rostosky et al., 2003; Edwards et al., 2008; Hull et al., 2011). The study also facilitates a better understanding of this mediation, showing that attendance and praying seem to work both through sexual permissiveness and through other pathways, while the possible influence of salience is fully mediated by sexual permissiveness.

First, the double causal path from attendance to sexual behavior (via attitudes and through other ways) has been found previously (Hull et al., 2011). This is easy to understand within the TRA. Attending religious services may clarify and reinforce the messages of a religion (Rostosky et al., 2004; Regnerus, 2007), and promote the internalization of values (Thornton and Camburn, 1989), which explains its impact through attitudes. However, it may also have additional ways of influence, such as providing social support for certain behaviors (Mott et al., 1996; Rostosky et al., 2004; Edwards et al., 2008, 2011), or even as a form of social control, both from parents and from peers (Regnerus, 2007).

Second, our results confirm previous studies which suggest that the relationship between religious salience and sexual initiation is mainly mediated by attitudes. This mediation had not been tested so far, but different studies had found associations between salience and attitudes (Jung, 2016), and between attitudes and sexual behavior (Edwards et al., 2008). Religious salience implies personal endorsing of the moral values of a religion, which usually include conservative sexual attitudes (Sheeran et al., 1993). However, one does not expect that having religious salience implies a specific social environment (unless attendance is also present). These connections make sense in the TRA framework (Fishbein and Ajzen, 2010).

### Limitations and Strengths

This study has several limitations. First, as our study was based on a cross-sectional design, an explanation of the results of mediation analyses must proceed with caution. For example, the relationship of sexual attitudes with sexual behavior could be bidirectional; that is, sexual attitudes can influence the adolescents’ sexual initiation, but an early sexual initiation could also foster sexual permissiveness. Similarly, we cannot assume a clear causal direction in the relationship between religiosity dimensions and sexual initiation. Although the exposure to a religious environment within the family or the school usually delays sexual initiation, the fact of having sexual relationships could lead to lower religiosity. The proposed model was confirmed using logistic regression and path analysis; however, future research should evaluate the causal path using a longitudinal design. Second, although it is not uncommon to study religiosity or attitudes through non-standardized questionnaires, this approach entails some limitations that future studies could address. Third, participants in this study were a sample of Christians (predominantly Catholics) and non-religious participants from four Spanish-speaking countries. Consequently, our sample limits the scope of the results’ generalization to populations that differ by religious affiliation. However, this fact increases the internal validity of the results. With a more heterogeneous sample, we could test whether the associations found are actually present only in one or more of the religions under study. In our case, the findings seem to be valid at least for Catholicism (the majority religion in Hispanic countries).

Despite these limitations, this study has notable strengths. First, this is the first study that tested how sexual attitudes mediate the effect of three religiosity dimensions on sexual behavior. To the best of our knowledge, few studies have tested a mediation model between a specific religiosity dimension and sexual behavior (Mott et al., 1996; Hull et al., 2011), and they tested one dimension only (attendance), while we added salience and prayer. Second, this was studied within a Hispanic context – an as of today insufficiently explored population. Third, the large sample permitted complex multivariate analyses, adjusting for different confounders.

## Conclusion

This study demonstrated that religiosity could be a relevant factor to explain sexual initiation during adolescence. In line with prior existing United States studies, but with a more detailed understanding of the issue, we found that sexual attitudes mediated this relationship in a sample of adolescents from Spanish-speaking countries. In addition, the relationship between religious attendance and prayer and sexual initiation is strong, independently of sexual permissiveness. Our study highlights that, in Spanish-speaking countries, which are predominantly Catholic, the fact of attending church and praying may greatly contribute to adolescents’ postponing sexual relations during adolescence, even independently of their attitudes on sexual permissiveness. Conversely, the effect of salience on sexual initiation seems to be fostered only through the mediation of sexual permissiveness. Our results point to an indirect effect of the three religiosity dimensions (and in particular, religious salience) through permissive attitudes. Educators, clinicians, and social agents should be sensitive to adolescents’ religious beliefs in order to enhance the effectiveness of sexual education programs.

## Data Availability Statement

The raw data supporting the conclusions of this article will be made available by the authors, without undue reservation.

## Ethics Statement

The studies involving human participants were reviewed and approved by Ethics Committee of the University of Navarra. Written informed consent from the participants’ legal guardian/next of kin was not required to participate in this study in accordance with the National Legislation and the Institutional Requirements.

## Author Contributions

JD, CL-dB, and MC contributed to conception and design of the study. MC organized the database. MC and AO performed the statistical analysis. CB and MC wrote the first draft of the manuscript. MR-G and AO wrote sections of the manuscript. All authors contributed to manuscript revision, read, and approved the submitted version.

## Conflict of Interest

The authors declare that the research was conducted in the absence of any commercial or financial relationships that could be construed as a potential conflict of interest.

## Publisher’s Note

All claims expressed in this article are solely those of the authors and do not necessarily represent those of their affiliated organizations, or those of the publisher, the editors and the reviewers. Any product that may be evaluated in this article, or claim that may be made by its manufacturer, is not guaranteed or endorsed by the publisher.

## Footnotes

1
http://proyectoyourlife.com/en/
